# How behavioural science can contribute to health partnerships: the case of The Change Exchange

**DOI:** 10.1186/s12992-017-0254-4

**Published:** 2017-06-12

**Authors:** Lucie M.T. Byrne-Davis, Eleanor R. Bull, Amy Burton, Nimarta Dharni, Fiona Gillison, Wendy Maltinsky, Corina Mason, Nisha Sharma, Christopher J. Armitage, Marie Johnston, Ged J. Byrne, Jo K. Hart

**Affiliations:** 1 University of Manchester, Stopford Building, Oxford Road, Manchester, M13 9PT UK; 2 Science Centre, Staffordshire University, Leek Road, Stoke-on-Trent, ST4 2DF UK; 3 Bradford Institute for Health Research, Duckworth Lane, Bradford, BD9 6RJ UK; 4 University of Bath, Claverton Down, Bath, BA2 7AY UK; 5 Inverness College University of the Highlands and Islands, 1 Inverness Campus, Inverness, IV2 5NA UK; 6 NHS Grampian, Public Health Directorate, 2 Eday Road, Aberdeen, AB15 6RE UK; 7 University of the West of England, Frenchay Campus, Coldharbour Lane, Bristol, BS16 1QY UK; 8 University of Aberdeen, Health Sciences Building, Foresterhill, Aberdeen, AB25 2ZD UK; 9 Health Education England, 3 Piccadilly Place, Manchester, M1 3BN UK

**Keywords:** Implementation science, Behaviour, Health partnerships

## Abstract

**Background:**

Health partnerships often use health professional training to change practice with the aim of improving quality of care. Interventions to change practice can learn from behavioural science and focus not only on improving the competence and capability of health professionals but also their opportunity and motivation to make changes in practice. We describe a project that used behavioural scientist volunteers to enable health partnerships to understand and use the theories, techniques and assessments of behavioural science.

**Case studies:**

This paper outlines how The Change Exchange, a collective of volunteer behavioural scientists, worked with health partnerships to strengthen their projects by translating behavioural science in situ. We describe three case studies in which behavioural scientists, embedded in health partnerships in Uganda, Sierra Leone and Mozambique, explored the behaviour change techniques used by educators, supported knowledge and skill development in behaviour change, monitored the impact of projects on psychological determinants of behaviour and made recommendations for future project developments.

**Discussion:**

Challenges in the work included having time and space for behavioural science in already very busy health partnership schedules and the difficulties in using certain methods in other cultures. Future work could explore other modes of translation and further develop methods to make them more culturally applicable.

**Conclusion:**

Behavioural scientists could translate behavioural science which was understood and used by the health partnerships to strengthen their project work.

## Background

The Tropical Health and Education Trust state that health partnerships, collaborations between high-income countries and low- and middle income countries (LMIC), “strengthen health systems through health service skills transfer and capacity development” [1]. Health partnerships have been a mainstay of capacity building in LIC. Training is often the go-to solution when changes in health professional practices are required, and our experience tells us that health partnerships are no different. Miller’s pyramid, well known in medical education [2], neatly shows the building blocks of practice from knowing and knowing how, through showing how and all the way to ‘does’. Educators typically assess the efficacy of their training through measures of knowledge and skill and sometimes by monitoring changes on ‘does’ through audit and/or impact on patient outcomes. Following a theory of change approach [3], Health Partnership projects are usually required to propose links from education, through changes in practice to impact on patient outcomes. However, there is little focus on *how* ‘shows how’ becomes ‘does’ Thus, the actual factors that determine whether ‘shows how’ ever becomes ‘does’ are typically not used to evaluate the efficacy of training or, more widely, the efficacy of health partnerships. We propose that examining change at this granular level has benefits for health partnership projects and in this paper, we describe The Change Exchange: a project in which nine behaviour change consultants were placed within four health partnerships. In it, we explore their activities, and the potential impact they could have through three case studies.

Although the focus of Health Partnerships is often on education and training, they do, of course, include techniques other than those to improve only knowledge and skills in their education. Many continuing professional development (CPD) activities present a rationale for people to change their practice or involve reflection on barriers to adopting the new practices. Implicit in these activities is an underlying theory of how behaviour changes. Although activities that target more than skills and knowledge are undoubtedly occurring, we have not yet explored whether partnerships could be more effective if behavioural science became more systematically and explicitly incorporated. Whether and in what circumstances knowledge and skills are translated into action has received considerable research attention in the fields of behavioural science, implementation science and their root science of psychology [4–6].

Behavioural science draws from a wide range of theories involving both conscious and unconscious processes but CPD activities, aimed at changing behaviour, tend to be restricted to addressing conscious, reflective thoughts and attitudes. Healthcare professional behaviour, like all human behaviour, is influenced by both types of process –not just what we believe but also our emotions, needs and habits [7, 8].

Behavioural scientists have developed the Behaviour Change Technique Taxonomy (BCTT) [9] which groups over 90 behaviour change methods into 16 types. These techniques might be useful for educators to identify or adopt in their training if they are to change practice of trainees.

The application of theory improves our ability to change behaviour [10]. This is because our ability to change behaviour relies on the intervention targeting the correct mechanism(s) of action. Theories of how behaviour changes include mechanisms of action and therefore our interventions become more focused on determinants of change. However, the complexity and sheer number of theories may limit both the likelihood that theory is applied. An early attempt at summarising and synthesising [11] may have increased the use of behavioural science theory in the implementation of evidence-based practice and a recent framework makes the main ideas even more accessible: behavioural influences can be thought about using the broad categories of capability, opportunity, and motivation (The COM-B framework) [12]. Capability includes knowledge and skills, opportunity includes physical opportunity (environment) and social opportunity (social pressure and norms), and motivation encompasses many aspects of explicit decision-making (e.g., weighing up pros and cons), as well as the influence of habit and automaticity in behaviour. The ‘B’ in the framework is ‘behaviour’. Although the many behaviour change theories (over 80 were found a recent review [10]) are complex and overlapping, the simplicity of the COM-B model provides educators with an opportunity to access behaviour change theory.

We would argue that there is efficacy and efficiency benefits from using more behavioural science theory in health partnerships. The consequences of a lack of engagement with behavioural science theories and methods has been a lack of explicit and systematic incorporation of behaviour change techniques (BCT) in education and a lack of monitoring the impact of education on the determinants of practice beyond competence. In other words, there has been an overt focus on the ‘C’ of the COM-B framework.

## Case studies

The Change Exchange is a project, funded by the Health Education England Global Health Exchange and the DFID funded Health Partnership Scheme and managed by the Tropical Health and Education Trust, with the remit of strengthening health partnerships by using behavioural science [13]. The project was developed after experiences of assisting a specific health partnership deliver and understand the impact of training in acute illness management in Uganda [14–17]. During that partnership, we proposed three ways that behavioural science could contribute to strengthening the activities of health partnerships, by reconceptualising training in terms of behaviours (the ‘behaviour’ of the COM-B) as opposed to knowledge and skills (the ‘capability’ of the COM-B). Firstly, we could enhance *interventions* by observing educational interactions, noting the BCTs used, and making recommendations on how to adapt existing content or add new BCTs that target opportunity and motivation, as well as capability, thus making behaviour change more likely. Secondly, we could offer *assessment methods* by tailoring questions to ask participants that would assess not only their capability but also their opportunity and motivation to perform specific tasks set out by the education and training, thus identifying barriers and facilitators to changes in practice that could be targeted, not only changes in knowledge and skills. Thirdly, we could facilitate *evaluations,* by building the capacity of health partners to engage in robust data collection for evaluation and research of their partnership, with a specific focus on changing practice. The following case studies will describe the implementation of these three: enhancing interventions, offering assessment methods and facilitating evaluations.

### Case example 1: Enhancing interventions and offering assessment methods in obstetric care in Masaka, Uganda

The aim of the health partnership between the Royal College of Obstetricians and Gynaecologists (RCOG) and Kitovu Hospital is to improve obstetric care and reduce the incidence of obstetric fistula in the Masaka region of Uganda by co-ordinating and delivering a training package (‘Excellence in: Obstetric Skills’). The course is a three-day programme of lectures, workshops and skills clinics incorporating a train-the-trainer model, to ensure the sustainability of the programme and the transfer and retention of skills from UK faculty to local health care professionals.

Pairs of behavioural consultants firstly observed, reviewed and coded the BCTs in both the training of health professionals, and in training new course facilitators, using the BCT Taxonomy [9]. The functions of these BCTs were then explored in terms of the COM-B model to identify potential gaps in the provision of behavioural support.

As expected for a skills training programme, many techniques were present to support capability (e.g., didactic teaching and providing opportunities to learn and practice skills). Some techniques to improve reflective *motivation* (e.g. verbal persuasion, setting positive outcome expectancies) and automatic processes (e.g., using mnemonics) were observed. However, there was limited techniques addressing types of motivation associated with sustained change (i.e., ‘autonomous’ motivation, which is based on one’s personal values, rather than facilitated through coercion (feeling one ‘should’) or external contingencies (rewards or penalties) [18]; and habit formation). Few techniques were observed to support physical and social *opportunities* for implementation of change to practice*.*

Observations of the course were supplemented by visits to health centres to observe trainees within their working environment, and focus groups with delegates from the course. These sources exemplified the importance of poor *opportunity* in limiting the implementation of changes in practice. This was evident both through limited environmental opportunities, including the lack of resources and basic equipment, opportunities for hands-on practice as a result of low levels of attendance at health centres by labouring women and limited CPD opportunities for healthcare workers. Similarly, consultants identified the lack of social opportunity as a factor limiting changes in practice, finding it hard to influence colleagues to bring about necessary changes in procedures.

We made recommendations to the RCOG team, based on the COM-B Framework [12], for changes to be implemented in the next iteration of the course. To target autonomous *motivation* we recommended to a) incorporate more examples of the benefits Ugandan trainees had found from changing their practice (i.e., presenting a locally relevant, meaningful rationale for change), and b) modify action planning activities to include personalised goals. To foster more automatic *motivation* (i.e., cue-response behaviours) we recommended the development of posters to be displayed in health centres acting as behavioural cues to action. To foster social *opportunity,* the use of social media platforms such as, Facebook and WhatsApp groups were recommended, which could be accessed from even the most remote areas. Finally, recommendations were made to embed education in behaviour change techniques explicitly into the materials for UK course facilitators and Ugandan trainers.

To action our final recommendation, we were invited by the RCOG to contribute behavioural science training to the train-the-trainer programme for both the UK and Ugandan professionals. Through the same set of activities of the training course (lectures, workshops and skills practice) training was provided on motivational support. Specifically, we addressed how motivational techniques could be used to motivate co-workers to change their practice and be implemented in outreach activities to encourage greater use of health centres by local women rather than receiving care from a local birthing attendant. Ugandan trainers were provided with instruction and mentoring on how to guide and motivate new trainees (e.g., provide critical feedback in a positive way). Overall, the inclusion of behavioural science led to improvements in the interventions within the education and training and also the inclusion of behavioural theories and techniques within the masters training project.

### Case example 2: assessment of capability, opportunity and motivation in mozambique and sierra leone

#### Mozambique

The Ipswich-Beira partnership aims to connect specialist health professionals in Ipswich Hospital Trust, UK and Beira Central Hospital, Mozambique to share expertise and offer practical assistance to improve hospital services for local people in Beira. One current focus of the partnership is medication safety, including implementing a revised inpatient prescription chart (known as a cardex in Mozambique). The partners had worked over several years to adjust the cardex to include medication safety features including a box to alert prescribers to allergies, pregnancy or other important information. However, it was unclear how widely the cardex was being used and ward staff views on its use had not been systematically sought or analysed.

We audited the use of the cardex across the 23 hospital wards: 6 had implemented the cardex and two were actively using it. We explored medical staff members’ perceptions of using the new chart through short, opportunistic, one-to-one interviews. Initially, we ascertained from ward nurses which cardex was routinely used and then asked open questions such as ‘what do you think of the new cardex?’ ‘How easy/difficult is it/would it be to use?’ ‘What would make it more/less likely for you to use the cardex?’ ‘In what way could it be improved?’ Following this, we grouped responses into themes using the overarching behavioural COM-B framework and made key recommendations.

From our analyses, physical capability and physical opportunity were key areas to target to facilitate the implementation of the new cardex. In terms of capability, some nurses felt unsure of how to complete the information required in the allergies box and of whose responsibility it was to sign the new cardex. We recommended that these be addressed through short ward-based practical training during the rollout of the new cardex led by a ‘credible source’ such as the nursing director, which would also recommend that staff seek social support from other staff if unsure. Staff reported that the new cardex took no additional time to complete, a key physical opportunity facilitator for implementation and for medication safety on busy acute wards [19]. However, most respondents felt that the cardex layout was an opportunity barrier to completion, important since practice change is more likely when the new behaviour is easy and attractive to adopt [20]. Therefore we recommended space-saving changes such as increased box heights, changing the numbering of days to prompt correct use of the cardex and reorganising and grouping medication types, the latter being since polypharmacy is associated with increased medication error rate [21]. Users and non-users of the new cardex alike appeared highly motivated to use the new cardex, reporting that it would improve patient safety and that the prompt words for allergies and pregnancy helped staff remember to ask these things.The findings and recommendations were highlighted in our interim report for all partners, as well as through a short presentation delivered to the Nursing Director who had requested this work, the Medical Director and other key stakeholders in Beira.

During a second partnership visit to Beira in November 2016, we repeated the auditing process visiting 17 wards (six had closed for building work since the previous visit). This time, four wards were actively using the new cardex.

In our return visit to the partnership in November 2016, no further versions of the cardex had been produced by the partnership. In a discussion with two Pharmacists leading this project, they advised that opportunity barriers had prevented this: time and budget shortages, but they looked forward to presenting the cardex at a conference next June with hopes that it would be adopted nationally. Although recommendations have not been implemented to date, the audit and interviews, feedback and recommendations framed around the COM-B framework, gave a clearer picture of the cardex implementation to the health partnership. The use of the COM-B framework ensured that the issue of implementation of the cardex system was viewed from the three perspectives of capability, opportunity and motivation. Therefore, taking a behavioural approach led to recommendations that future interventions, to improve cardex system use, should focus on all three areas i.e., go beyond training heathcare professionals to use the system and look at the implementation in terms of how the systems encourage or discourage healthcare professionals to use the cardex.

### Sierra Leone

The partnership between Plymouth University Peninsula Schools of Medicine and Dentistry (PUPSMD) and Masanga, Sierra Leone, aims to improve the resilience of the people of Sierra Leone towards outbreaks of highly infectious diseases, including Ebola. The partnership uses virtual learning and computer gaming technology to deliver education and training to healthcare professionals and community members, regarding the steps to take if a highly infectious disease is suspected or found in a family member or friend. Due to the training being delivered via a tablet device, we could work with the partnership to design questionnaires that would be delivered either before or after the training, on the same tablet device. The questionnaire assessed the capability, opportunity and motivation of the healthcare workers and community members to do the behaviours required of them, as per the training. We cluster randomised groups of people undergoing the training so that some of them received the questionnaire before training and some afterwards. We were then able to compare those two groups and could draw inferences about the impact of the training on expected behaviours and determinants of behaviours i.e., capability, opportunity and motivation. We found that healthcare professionals found it difficult to answer Likert response scales (scales of 1 to 7 with 1 indicating strongly disagree to 7 indicating strongly agree). This led to us training a partnership team member to conduct focus groups, so that she could explore the capability, opportunity and motivation barriers to the specific desired behaviours in more depth with the healthcare professionals. The results of the questionnaires and focus groups will be reported elsewhere, by the partnership team.

The behiavoural approach taken meant that both health partnerships learnt more about determinants of practice, we could asses these and they were able to feed that information back into the development of their education and training and into evaluation of their project work.

### Case example 3: knowledge and skills for behaviour change evaluation in Uganda

Our final case study focuses on the MOMENTUM project: a health partnership between the Royal College of Midwives (RCM) and the Ugandan Private Midwives’ Association (UPMA). Momentum was developed in response to two pressing needs; the high maternal and neonatal mortality rates in Uganda; and the outcome of the Global Midwifery Twinning Project [22]. The Global Midwifery Twinning Project identified the need to develop national standards for learning and assessment in practice, support midwives to improve their mentorship skills and develop a work-based learning module to prepare midwives for mentorship. To address these goals, the RCM and UPMA jointly delivered a 20 month project to develop a model of MENTorship for Ugandan Midwifery (MOMENTUM). Training in Uganda was delivered at the start of the project, with two further workshops spaced roughly six months apart. In addition, seven Ugandan midwives who were acting as mentors to student midwives were twinned with UK midwives for knowledge, skill and mentoring support. Our objective was to establish how health behaviour change theory could enhance the impact and sustainability of the project.

Four behavioural consultants undertook three visits (two on the first and a further two on the second and third) to Uganda in January, June and November 2016. In the first visit, the consultants introduced, to the RCM and the UPMA, the COM-B, the importance of a behavioural approach to health professional practice change and started to develop the underpinning relationships between behavioural scientists and the partnership team. The second visit aimed to establish how behaviour change theory could be useful to the project’s aims of maximising the training of student midwives through mentoring. Drawing on the Theoretical Domains Framework (TDF) [23, 24], our observations, interviews and discussion groups, and visits to two contrasting midwifery settings, we gleaned an understanding of the behaviour changes that had been experienced as part of the project, and what future changes were anticipated and the behavioural determinants of those changes. It was apparent that the project drew on several of the constructs of the TDF with greater emphasis on social support, which related to both social opportunity (believing that people want you to engage in particular behaviours) and reflective motivation (desires to make practice changes), within the COM-B framework. Furthermore, midwives appeared more competent in their mentoring roles and providing more optimal learning environments for students.

The original proposal for the MOMENTUM project included a plan to undertake a substantive piece of research underpinned by the principles of action research methodology. However, the action research approach was no longer feasible amongst the prioritisation of key project activities and maintaining milestones. Through collaborative discussions with the partnership team, we could help identify a feasible study design and appropriate research questions alongside considerations of skills and knowledge inherent in the team for conducting the research. Although this was not assistance that could only be provided by behavioural scientists, the knowledge of mixed methods research and psychological theories underpinning mentoring, meant that the behavioural scientists could adapt to the local needs of the partnership, providing research support.

Our observations and initial reflections from the interviews indicated that the training workshops, coupled with the twinning and mentoring components of the project, appeared to be instrumental in strengthening both key mentoring skills and a sense of competency in using these skills in practice i.e., capability. Our rapid review of the literature highlighted the importance of mentoring programmes in facilitating the self-efficacy of student midwifes [25]. Self-efficacy is a person’s belief in their ability to do a particular task or succeed in a particular goal [26]. It was possible that an increase in the self-efficacy of mentors may have been an unanticipated outcome from this project and one that could warrant some further exploration. We therefore recommended a qualitative study exploring the impact of participating in the MOMEMTUM project on mentors’ and students’ self-efficacy would be a valuable addition to the literature on midwifery mentoring in LMICs.

Further email discussions and Skype calls with the health partnership in the period after our visit focused on agreeing roles and responsibilities, a plan for obtaining ethical approval, and the training needs of the UPMA team to collect the data. With the study design and skillset of the team in mind, we returned to Uganda to deliver a one week research skills training workshop. Workshops explored literature searches, conducting focus groups, self-efficacy, timelines and milestones. We worked collaboratively throughout, reviewing questions and approaches to data collection methodology (in this case focus groups) that were designed to be consistent with the literatures on self-efficacy and mentoring but also appropriate for Ugandan culture, for example, incorporating the use of images and analogies that we had previously observed to be very successful in crossing both professional and cultural boundaries during the training sessions with midwives. Whilst there are no further visits planed, we continue to support our colleagues at the UPMA with aspects of data collection and analysis as well as their own professional development as researchers.

Our experiences of developing evaluations in our health partnership brought into sharp relief the cultural assumptions that bind many of our theories, constructs, measures and methods for data collection. It reminded us of the cultural differences that exist in the UK and why we should be cognisant of these when we undertake any research or discussions. The inclusion of behavioural scientists in this project had two interwoven outcomes. Firstly, the behavioural scientists, with their expertise in science methods and teaching and training, were able to build capacity locally in research knowledge and skills. Secondly, they were able to guide the generation of research questions such that the findings will build on what is already known about self-efficacy and mentoring. These two outcomes are beneficial to HP in empowering the LMIC partner to take a lead in the research around HP and also ensuring that the research asks questions that build on previous research.

## Discussion

Our work with health partnerships has highlighted a need for more focus on the determinants of practice in the design and evaluation of partnership projects. We have found that this focus can be provided by volunteer behavioural scientists, working alongside and embedded within the partnerships. We have shown that small projects can be embedded within the larger partnership that elucidate ways in which partnerships can be strengthened and sustained and that, in some cases, these small projects can themselves form research studies. Additionally, we have illustrated that partnership teams can benefit from capacity building exercises, making the use of behavioural theories and methods sustainable within the partnerships beyond the involvement of the behavioural scientists.

There are many ways in which this initial work could be taken forward. The Change Exchange was a pilot of a method of engaging behavioural scientists in volunteering activities and we certainly found that there were many volunteers who wanted the opportunity to contribute and learn within health partnerships. Moving from pilot to a routine part of health partnerships could have benefits for the content of training courses, and the knowledge and skills of behaviour change of both UK and local health care professionals, and the development of an evidence base. Firstly, reconceptualising training in terms of behaviours as opposed to knowledge and skills is crucial, particularly in understanding how the context in which a person works will inevitably impact on their activities. Secondly, systematically varying or adding behaviour change techniques within and across health partnerships could provide some evidence about how effective behaviour change interventions are in changing practice, and doing this within either a complex interventions [27] or natural experiment [28] framework would increase the robustness. Finally, assessing the theoretical determinants of change before training would mean that techniques could be selected to address the determinants shown to be a challenge. For example, behaviour change techniques designed to increase motivation would be ineffective if the healthcare professionals were already motivated, but improved planning might be a useful alternative [29]. Assessing before and after means that educators can understand how the training is affecting the internal world of the trainee.

### Limitations

The work was not without its challenges. The educators, although very receptive to behavioural approaches, already had full agendas for their education and training visits. This made it difficult to find time and space in training curricula to make changes. It would be beneficial, in future projects, for behavioural science to feature at the start and throughout the project. The projects move at a pace which was unfamiliar to the behavioural scientists, who were used to a slower pace in academic life. Further work will map the competencies required for this type of work against those developed through the training in behavioural science afforded by health psychology and other disciplines. In terms of sustainability and equity, we found it challenging to identify and collaborate with behavioural scientists based in the countries in which the projects were active. Building capacity and identifying potential behavioural science experts in each country of the partnerships would be beneficial in terms of both equity and sustainability of these types of activities. Finally, the measures, methods and theories brought by the behavioural scientists have been developed largely through research with the so-called WEIRD (Western Educated Industrialised Rich and Democratic) population samples [30]. It is both a challenge and an opportunity to work within partnerships to test and advance the science of behaviour itself, resulting in a mutually beneficial collaborative effort. Our case studies show that health partnerships perceive a benefit of the inclusion of behavioural science and we are able to conclude that using behavioural science in this way was feasible and acceptable. Further research in which behavioural science was robustly evaluated against other approaches would be required to make firm conclusions about the degree of added value.

## Conclusion

The Change Exchange is an example of how behavioural science can be translated in situ to support health partnership work. There are challenges to the translation of behavioural science into health partnerships in this manner including having time and space and the cultural appropriateness of theories and methods from high-income country science. Future work of The Change Exchange will tackle these issues and build partnerships with LMIC researchers with behavioural science expertise.

## Abbreviations

BCT: Behaviour change technique
COM-B: the Capability, Opportunity, Motivation, Behaviour Framework
CPD: Continuing professional development
RCM: the Royal College of Midwives
RCOG: the Royal College of Obstetricians and Gynaecologists
TDF: the Theoretical Domains Framework
UPMA: the Ugandan Private Midwives’ Association
WEIRD: Western, educated, industrialised, rich and democratic
